# Detection of HIV-1 Neutralizing Antibodies in a Human CD4^+^/CXCR4^+^/CCR5^+^ T-Lymphoblastoid Cell Assay System

**DOI:** 10.1371/journal.pone.0077756

**Published:** 2013-11-28

**Authors:** Robert J. McLinden, Celia C. LaBranche, Agnès-Laurence Chenine, Victoria R. Polonis, Michael A. Eller, Lindsay Wieczorek, Christina Ochsenbauer, John C. Kappes, Stephen Perfetto, David C. Montefiori, Nelson L. Michael, Jerome H. Kim

**Affiliations:** 1 Military HIV-1 Research Program, WRAIR, Silver Spring, Maryland, United States of America; 2 Department of Surgery, Duke U. Medical Center, Durham, North Carolina, United States of America; 3 Department of Medicine, University of Alabama at Birmingham, Birmingham, Alabama, United States of America; 4 Birmingham Veterans Affairs Medical Center, Research Service, Birmingham, Alabama, United States of America; 5 Vaccine Research Center, NIH, Bethesda, Maryland, United States of America; George Mason University, United States of America

## Abstract

Sensitive assays are needed to meaningfully assess low levels of neutralizing antibodies (NAbs) that may be important for protection against the acquisition of HIV-1 infection in vaccine recipients. The current assay of choice uses a non-lymphoid cell line (TZM-bl) that may lack sensitivity owing to over expression of CD4 and CCR5. We used transfection of a human CD4+/CXCR4+/α_4_β_7_+ T-lymphoblastoid cell line (A3.01) with a CMV IE promoter-driven CCR5neo vector to stably express CCR5. The resulting line, designated A3R5, is permissive to a wide range of CCR5-tropic circulating strains of HIV-1, including HIV-1 molecular clones containing a Tat-inducible *Renilla* luciferase reporter gene and expressing multiple Env subtypes. Flow cytometric analysis found CCR5 surface expression on A3R5 cells to be markedly less than TZM-bl but similar to CD3.8 stimulated PBMC. More importantly, neutralization mediated by a diverse panel of monoclonal antibodies, HIV-1 positive polyclonal sera and sCD4 was consistently greater in A3R5 compared to TZM-bl cells. The A3R5 cell line provides a novel approach to guide the development and qualification of promising new HIV-1 vaccine immunogens.

## Introduction

Modest protection against acquisition of HIV-1 was observed in a recent phase III clinical trial (RV144) of ALVAC-HIV and AIDSVAX B/E in Thailand [1]. The vaccine combination generated low levels of primarily tier 1, type-specific NAbs measured in the TZM-bl or T-cell line adapted assays[2,3]. These vaccine-induced antibodies were not identified as correlates of risk in RV144[4]. Nonetheless, broadly cross-reactive, potent neutralizing antibodies (bNAbs) may be an important consideration in future vaccine design [5,6]. Results of passive immunization studies in non-human primates [7–9] and the ability of NAbs to exert strong selection pressure on the virus in HIV-1-infected individuals support this proposition. However, NAbs induced by candidate HIV vaccines have typically proven weak, especially against circulating or transmitted strains of the virus[10–14]. The uncertainty surrounding the magnitude of neutralization necessary for protection in humans requires that vaccine induced NAb activity be accurately quantified by the most sensitive *in vitro* assays available[15,16]

A variety of assay platforms have been used to assess NAb responses against HIV-1[14,17–20]. Among these, genetically engineered cells lines in combination with Tat-inducible luciferase (Luc) reporter genes have been extremely valuable for studies of HIV-1 neutralization and escape [21–23], the identification of HIV-1-infected subjects who possess broadly NAbs [24–27], the identification and characterization of broadly neutralizing mAbs [28–35] and the mapping of epitopes of autologous NAbs [36–43] and bNAb [32,44–50] in sera from HIV-1 infected subjects. The two most prevalent cell lines are TZM-bl[23,51] (HeLa derivative, human epithelial origin) and U87.CD4.CCR5 cells (human astroglioma cell line)[21,22]. However, evidence from several studies suggest that TZM-bl cells may not support the detection of neutralizing antibodies to certain epitopes, possibly owing to artificially high surface expression of CD4 and CCR5[52–54]. The observation that TZM-bl and U87.CD4.CCR5 cells exhibit similar levels of sensitivity[55] indicate limitations may exist for the latter assay as well. Here we describe a CD4+/CXCR4+/α_4_β_7_+/CCR5+ T-cell line, A3R5.7 (designated A3R5), that supports the detection of HIV-1-specific neutralization by mAbs, sCD4 and polyclonal plasma from multiple subtypes encompassing a range of epitopes on the HIV-1 envelope with sensitivity similar to or greater than that observed in the TZM-bl line.

## Materials and Methods

### Cloning of pCMV-CCR5neo

pCMV-CCR5neo consists of the ccr5 gene (nt positions 240 to 1298) amplified by PCR from PBMC DNA and inserted into the pCR3.1 expression vector (Invitrogen, Carlsbad, CA) downstream of the CMV immediate early (IE) promoter containing the neomycin phosphotransferase gene as a selectable marker. The PCR primers used to generate this fragment were: CCR5-1, 5’-ggtggaacaagatggattat-3’ and CCR5-2, 5’-catgtggcacaactctgactgg-3’.

### Generation of CCR5 expressing T-cell lines

Fifteen million cells of the CD4+/CXCR4+ human lymphoblastoid T-cell line, A3.01, (NIH AIDS Research and Reference Reagent Program, Rockville, MD.) were subjected to electroporation (Bio-Rad, Hercules, CA.) with 40µg of the pCMV-CCR5neo construct followed by resuspension in RPMI medium (Gemini Bio-Products, West Sacramento, CA) supplemented with 15% fetal bovine serum (Gemini Bio-Products), 1% L-glutamine and 1% penicillin/streptomycin (Gemini Bio-Products) (cRPMI) for 48 hours in a humidified 37ºC/5% CO_2_ chamber[56,57]. Subsequently, the cells were washed and transferred to cRPMI medium supplemented with 600 µg/ml (active) geneticin sulfate (G418) (Sigma, St. Louis, MO) and incubated for three weeks to select neomycin resistant cells. The resulting line was designated A3R5. Transfected cells were stained with a phycoerythrin(PE)-conjugated anti-CCR5 monoclonal antibody (clone 2D7; BD Biosciences, San Jose, CA) and analyzed by flow cytometry. Flow-Jo v9.5.3 (Treestar, Ashland, OR) was used to determine frequency of expression and median fluorescence intensity (MFI). Antibody binding capacity (ABC), a correlate to the number of antigens expressed on the cell surface was quantified using Quantum™Simply Cellular® Beads as per the manufacturers suggested procedure (Bangs Laboratories, Fishers, IN)[58]. Post-selection CCR5 receptor density was found to be significantly lower than that observed on stimulated peripheral blood mononuclear cells (PBMC). Subsequently, fluorescence activated cell sorting (FACS) (Coulter Epics, Coulter, Hialeah, FL) was employed to isolate cell populations with the highest (upper 25%) CCR5-receptor number. Cells were then expanded in the presence of G418. The first sort was designated A3R5.1. Sorting was repeated eight times, generating lines A3R5.2 through A3R5.9.

A3R5.7 cells were expanded and analyzed for expression of CD4, CCR5 and α_4_β_7_ on a LSR II flow cytometer (BD Biosciences, Boston, MA). The cells were surface stained with a panel of fluorochrome-conjugated mAbs to CD4(PE;clone RPA-T4;BD Biosciences), CCR5(PE;clone 2D7;BD Biosciences) and α_4_β_7_(APC;clone ACT 1;NIH AIDS Research and Reference Reagent Program as contributed by A.A. Ansari) [59] prior to fixation with 2% paraformaldehyde. The α_4_β_7_ ACT1 clone was conjugated to allophycocyanin (APC) using the Lightning-Link APC-XL Conjugation kit (Innova Biosciences, Cambridge, UK). All experiments were performed using matched isotype controls. Receptor density testing was repeated with Quantum Simply Cellular beads. Expression of α_4_β_7_ was initially monitored in the presence and absence of 10nM retinoic acid (Sigma). The addition of retinoic acid did not result in significant enhancement of α_4_β_7_ surface expression (data not shown) and was not included in experiments constituting the final analyzed dataset. 

### Other cells

293T cells were purchased from the American Type Tissue Culture Collection (ATCC, Manassas, Va.). Human TZM-bl and CEM.NKR-R5-Luc cells were obtained from the NIH AIDS Research and Reference Reagent Program as contributed by John Kappes and Xiaoyun Wu and Drs. John Moore and Catherine Splenehauer, respectively [51,60–63]. SupT1.CCR5 cells were a kind donation by Dr. James Hoxie[64]. TZM-bl were grown in DMEM medium supplemented with 10% fetal bovine serum, 1% L-glutamine and 1% penicillin/streptomycin. CEM.NKR-R5-Luc was grown in RPMI medium supplemented with 10% fetal bovine serum, 4mM L-glutamine and 1% penicillin/streptomycin and 0.8 mg/mL G418. SupT1.CCR5 were grown in RPMI medium supplemented with 10% fetal bovine serum, 4mM L-glutamine and 1% penicillin/streptomycin and 0.2 ug/ml Puromycin. HIV-1 negative PBMC from a single donor were isolated by standard Ficoll method, stimulated with either CD3/CD8 bi-specific antibody (NIH AIDS Research and Reference Reagent Program)[65,66] as contributed by Johnson Wong and Galit Alter and 50U/mL rhIL-2 (Roche, Indianapolis, IN) or PHA/rhIL-2. PBMC were propagated in cRPMI with appropriate rhIL-2 for 72-96 hours prior to use in cell surface receptor expression and downstream infectivity/neutralization studies.

### Human Use

PBMC were collected by leukapheresis from a single HIV-1 negative donor as per the RV229c/WRAIR#1386 Protocol, which has been reviewed and approved by the Walter Reed Army Institute of Research (WRAIR) Institutional Review Board. Written informed consent was obtained under a procedure approved by the WRAIR institutional review board (IRB). 

### Monoclonal antibodies (MAbs), sCD4 and HIV+ plasmas

Monoclonal antibodies b12, 2G12, 2F5 and 4E10 were obtained from Polymum Scientific GmbH (Vienna, Austria). sCD4 was purchased from Progenics Pharmaceuticals (Tarrytown, NY). Monoclonal antibodies PG9 and VRC01 were obtained through the NIH AIDS Research and Reference Reagent Program[29,32]. Plasmas from CRF01_AE-infected individuals (T502281, T500617, T293735, T614109, T502102, T503006, T535902, T504258, T357545, T509989 and T518020) were obtained from Dr. Rheugpung at Siriraj Hospital(Bangkok, Thailand). This study utilized pre-existing, de-identified specimens collected under the approval of the Siriraj Ethics Committee (Bangkok, Thailand). The data were analyzed anonymously. Subtypes B, AE and C pooled seras/plasmas were previously described[54]. AIP3441, previously described in the context of a pooled reagent, is a Thai AE seropositive chronic plasma from the Royal Thai Army through the Thai Army Institute of Pathology in Bangkok and was kindly contributed by Dr. Nicos Karasavvas[67,68]. Written informed consent was obtained from this subject. HIV subtype for all samples was confirmed by full-length *env* gene sequencing (data not shown). Normal human serum (NHS) was purchased from Gemini Bio-Products (West Sacramento, CA) and used as a non-specific negative control for HIV-1 sera/plasma. All serum and plasma samples were stored at -80°C and heat-inactivated at 56°C for 1 hour prior to assay. Intravenous immunoglobulin (IVIG) is a pooled, polyvalent, IgG purchased from Bayer Healthcare, LLC (Clayton, NC) and used as a non-specific negative control for HIV-1 monoclonal antibodies.

### Virus stocks

The uncloned R5-tropic HIV-1 subtype B isolate US1 was obtained from the NIH AIDS Reference and Reagent Program as contributed by Nelson Michael [69] and expanded in PHA/IL-2 stimulated PBMC as previously described[11,70]. PBMC derived US1 was grown in various A3R5 cell lines in the presence of 10 μg/mL Polybrene (Sigma, St. Louis, MO) for up to fourteen days. Supernatants were harvested, lysed and assayed for HIV-1 core antigen p24 by sandwich ELISA according to the manufacturer’s protocol (Coulter, Hialeah, FL). 

Renilla luciferase (LucR) expressing replication-competent infectious molecular clones (IMC) expressing heterologous env genes from different HIV-1 clades are referred collectively in this study as IMC.LucR. The IMC.LucR include the IMC.LucR-Env constructs expressing an entire heterologous gp160, and the IMC.LucR-Env.ecto constructs where only the gp120 and the ectodomain of gp41 of the heterologous Env are expressed. All constructs express the cassette, LucR.T2A, inserted between the Env and Nef genes but the HIV-1 backbone varied: all IMC.LucR-Env.ecto used in this study are derived from subtype B HIV-1 NL4-3, and will be referred to in this publication as NL.LucR-Env.ecto [71,72], IMC.LucR-Env derived from either subtype C ETH2220.11B or CRF01_E CM235.2 will be denoted as ETH2220.LucR-Env and CM235.LucR-Env, respectively [73]. 

Env gene sequences from subtypes B, C and CRF01_AE were shuttled into the reporter virus backbone essentially as described[71]; details of genetic and some phenotypic properties of the resulting available panel of LucR reporter viruses, including those used here, will be reported elsewhere (Edmonds et al., manuscript in preparation). All constructs were isogenic in all HIV open reading frames other than env; env genes were from transmitted/founder as well as chronic HIV-1 strains[2,71,72]. Additional construct information including alternate nomenclature is shown in Table S1.

### Neutralization assays

For data shown in the third and fifth figures, neutralization was measured in 96-well culture plates by using Tat-regulated Firefly Luc and Renilla Luc reporter gene expression to quantify reductions in virus infection in TZM-bl and A3R5 cells, respectively. In other experiments, we determined that the neutralization sensitivity of IMC.LucR viruses in TZM-bl cells is the same whether the Firefly or Renilla reporters are used for detection of infection (data not shown). Neutralization in the TZM-bl assay was performed as described previously [74].

Neutralization assays in A3R5 cells were performed by incubating IMC.LucR virus (~ 50,000 relative light unit equivalents based on 4-day titrations of each virus performed in A3R5 cells) with serial 3-fold dilutions of test sample in duplicate in a total volume of 150 μl for 1 hr at 37°C in 96-well flat-bottom culture plates. Exponentially growing cells (90,000 cells in 100 μl of growth medium containing 25 μg/ml DEAE dextran hydrochloride) were added to each well. To avoid cell toxicity, the DEAE dextran (average molecular weight 500,000; Sigma Chemical Company, St Louis, MO) was added to the cell suspension immediately before the cells were dispensed to 96-well plates. One set of control wells received cells + virus (virus control) and another set received cells only (background control). After a 4-day incubation at 37°C, 100 μl of cell suspension was transferred to 96-well black solid plates (Costar) for measurements of luminescence using ViviRen Live Cell Substrate as described by the supplier (Promega).

Neutralization titers are the sample dilution (or concentration in the case of sCD4 and mAbs) at which relative luminescence units (RLU) were reduced by 50% compared to virus control wells after subtraction of background RLUs. For data shown in the fourth figure, 25 μL of 2-fold serially diluted MAb (25-0.78 μg/mL), sCD4 (25-0.78 μg/mL), pooled sera/plasma (1:40-1:40,960) or individual plasma (1:40-1:40,960) was incubated with an equal volume of appropriately diluted NL.LucR-CM235.ecto in a 96-well flat-bottom black luminescence plate (PerkinElmer) for 1 hour at 37^o^C/5% CO_2_. The final virus input was based on the dilution at which the mean RLU value in the virus only wells was 100-fold above the cell only background. Six wells each were reserved for background control (cells only), virus control (virus only) and non-specific inhibitor controls at a single 1:20 dilution (NHS and IVIG).

In A3R5.7 and PBMC assays, 50 μL of cells at 2 x 10^6^ cells/mL were added to each well. For TZM-bl assays, 50 μL of cells were pre-plated at 1 x 10^4^ cells/well 24 hours prior to infection. DEAE-dextran was added to all wells at a final concentration of 15 μg/mL. Plates were incubated for 72 hours at 37^o^C/5% CO_2_. Renilla-Glo substrate was diluted 1:100 per manufacturer’s instructions (Promega, Madison, WI) and 50 μL added to each well under low-light conditions. Plates were wrapped in foil and incubated at room temperature (RT) for 10 minutes prior to reading (1 second read per well) on a PE Victor X-light luminometer (PerkinElmer, Boston, MA).

Virus replication was measured as relative luminescence units (RLUs). Virus and antibody dilutions do not include volume of the cells. Neutralization titers were calculated as the sample dilution where RLUs were 50% of virus control wells. All samples were performed in triplicate in a minimum of two separate experiments. 

### Statistical analysis

Comparison of IMC.LucR titrations in A3R5.6 and A3R5.7 were performed using the paired t-test. Inhibitory concentration (IC_50_) neutralization titers were compared between the A3R5 cell lines and TZM-bl by combining all inhibitor/IMC pairs using the Mann-Whitney non-parametric test. In the fourth figure, individual inhibitors were compared between cell lines using the unpaired t-test with Welch correction. In the fifth figure, mean geometric 50% inhibitory concentration (IC_50_) and inhibitory dose (ID_50_) neutralization titers (GMT) were compared between the cell lines using the Wilcoxon Rank Sum test. All p-values are two-sided. Statistical analysis was performed with Prism 6 (Graphpad Software, La Jolla, CA). 

## Results

### Generation of A3R5 Cell Lines

**Figure 1:**
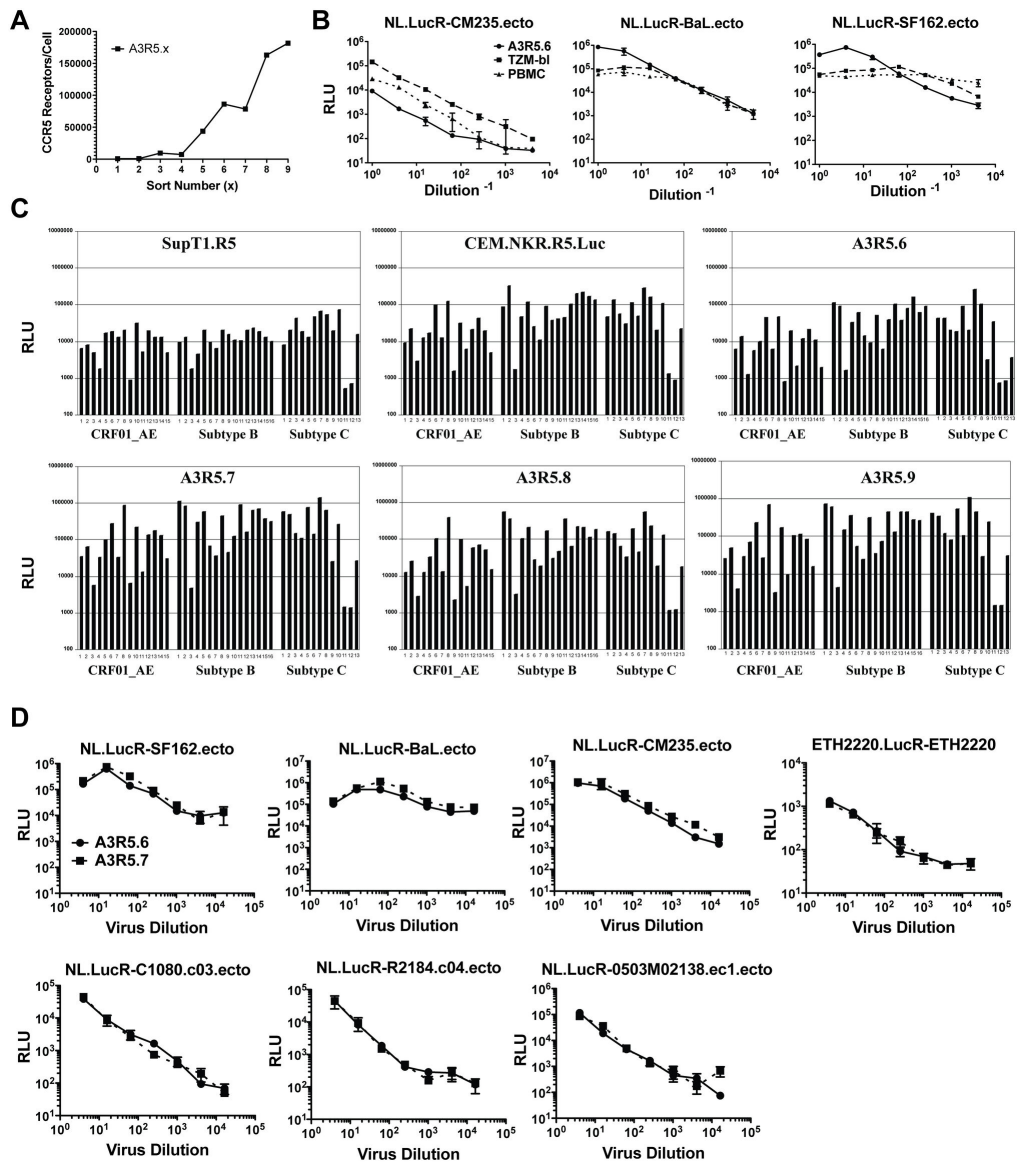
Initial characterization of A3R5 cell lines. **A. CCR5 surface receptor number in sequentially sorted A3R5 cell lines.** For each sort, 2.0 x 10^7^ cells were subjected to fluorescence activated cell sorting with the brightest population (top 25%) selected for expansion. Receptor number was calculated using Simply Cellular Beads. In general, each successive sort resulted in increasing CCR5 intensity on the cell surface. **B. Titration of NL.LucR-env.ecto in A3R5.6, TZM-bl and PHA/IL2 PBMC.** Replication competent reporter viruses encoding subtype B env (NL-LucR.T2A-SF162.ecto, NL-LucR.T2A-BaL.ecto) and subtype AE env (NL-LucR.T2A-CM235.ecto) were titered in each target cell. Background luciferase activity (cell control wells) was subtracted prior to plotting. The data show similar virus growth in the three target cells. **C. Infectivity of IMC.LucR viruses from multiple subtypes in various CCR5-expressing cell lines.** IMC.LucR from subtypes B, A/E and C were screened at a 1:3 dilution in triplicate for their ability to infect four different sorts of A3R5 cells as well as the SupT1.CCR5 and CEM.NKR.CCR5.Luc cell lines. The average relative light unit (RLU) values are indicated. IMC described in Table S1. While all cell lines supported IMC.LucR replication, infection was determined to be optimal in the A3R5.7 cell line. **D. Titration of IMC.LucR in A3R5.6 and A3R5.7 cells.** Replication competent reporter viruses encoding subtype B Env IMC (NL.LucR-SF162.ecto and NL.LucR-BaL.ecto) and subtype CRF_01 AE Env IMC (CM235, C1080, 0503M02138 and R2184 with backbone: NL.LucR-env.ecto) and subtype C Env IMC (ETH2220.LucR-ETH2220) were titered in the presence of DEAE-Dextran. Virus replication was measured as relative luminescence units (RLUs). Background luciferase activity (cell control wells) was similar in both cell lines and subtracted prior to plotting. Data represents the mean of three replicates with standard error bars (SEM). The data indicate no significant difference in IMC.LucR growth between the A3R5.6 and A3R5.7 cell lines (paired t-test;p0.5).

The A3R5 cell line was derived from transfection of A3.01, a CD4+/CXCR4+ T-lymphoblastoid cell line, with a vector containing the CMV IE promoter driven human CCR5 and bacterial neomycin resistance genes (pCMV-CCR5neo). Repeated flow cytometric sorting of geneticin (G418) selected cell populations with a PE-conjugated CCR5 monoclonal antibody resulted in nine polyclonal cell lines with progressive enhancement of CCR5 expression on the cell surface (Figure 1A). 

### Characterization of A3R5 Cell Lines

Initial infectivity studies with an uncloned subtype B R5-tropic primary isolate (US1) showed optimal p24 antigen production in the A3R5.6 cell line (Figure S1). Similar to that observed in TZM-bl cells and PHA/IL2 stimulated PBMC, titration of NL.LucR-Env.ecto viruses expressing Env proteins from subtype B HIV-1 (NL.LucR-SF162.ecto and NL.LucR-BaL.ecto) and CRF01_AE HIV-1 (NL.LucR-CM235.ecto), respectively, showed virus replication 1log_10_ over background at dilutions exceeding 1:1000 in the A3R5.6 line as measured by levels of virus-encoded LucR reporter gene expression 48 hours post infection (Figure 1B). Previous neutralization studies utilizing the A3R5-6 target cells in conjunction with a p24 antigen output (ELISA) assay system had supported multiple phase II vaccine studies [3,75,76]. Next, several A3R5 lines (A3R5.6 through A3R5.9) and two previously described R5-expressing T-cell lines were infected with a panel of IMC.LucR viruses encoding acute and chronic tier 1 and tier 2 Envs representing three diverse HIV-1 subtypes (CRF01_AE, B and C)(Figure 1C and Table S1). The data show that the A3R5 cell lines were permissive to all IMC tested, though to varying degrees which likely were a function of the respective Env genes expressed[71] (Chenine et al., manuscript in press) with optimal infection observed in the A3R5.7 line. Titration of IMC.LucR representing multiple subtypes showed no significant difference in permissiveness between the A3R5.6 and A3R5.7 cell lines (Figure 1D)(paired t-test; p>0.10), but overall higher RLU were reached in A3R5.7 cells, a desirable characteristic. 

**Figure 2:**
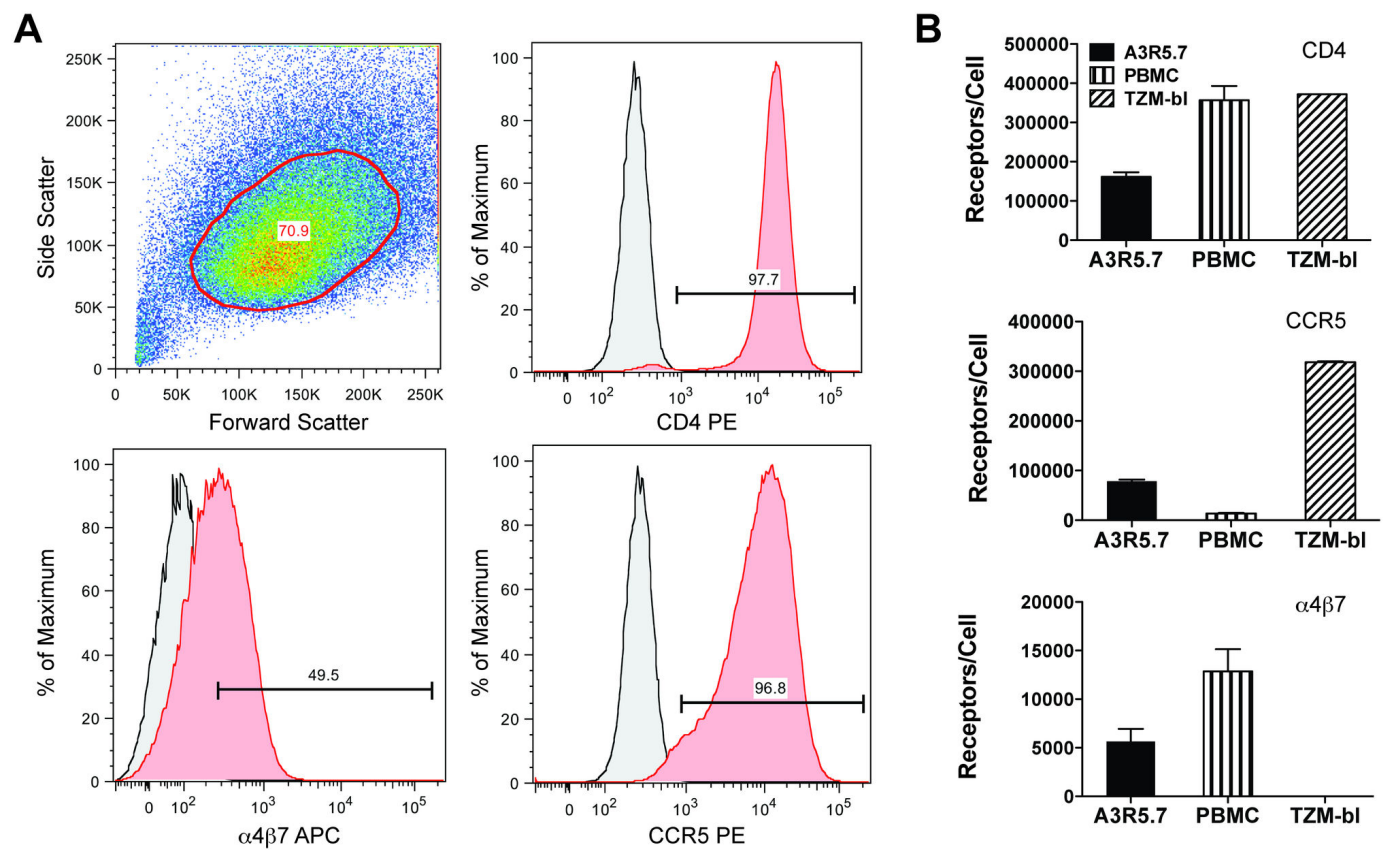
Receptor expression in A3R5.7 cells. **A. Flow cytometric analysis of CD4, CCR5 and α_4_β_7_ expression in the A3R5.7 cell line.** 0.5 x 10^6^ cells were singly stained for 30 minutes with fluorochrome-conjugated antibodies as shown followed by fixation in 2% paraformaldehyde. Data are representative of at least two independent experiments. Isotype controls are shown in grey. Nearly all cells were positive for CD4 and CCR5 while approximately half were positive for α_4_β_7_. **B. Comparison of cell surface CD4, CCR5 and α_4_β_7_ receptor densities in various cell targets.** 0.5 x 10^6^ cells were stained with fluorochrome-conjugated antibodies and compared to defined populations of similarly stained Quantum Simply Cellular beads. PBMC were stimulated with CD3.8 bi-specific antibody in the presence of 50U/mL rhIL-2. Assuming monovalent antibody-to-surface receptor binding, the Antibody Binding Capacity (ABC) calculated is equivalent to receptors/cell. Data represents the mean of two separate experiments. TZM-bl cells express high levels of CD4 and CCR5 but are negative for α_4_β_7_ while A3R5.7 cells possess CCR5 and α_4_β_7_ densities more similar to PBMC. CD4 expression on TZM-bl was beyond assay range.

Flow cytometric analysis found A3R5.7 cells to be positive for both CD4 (97.7%) and CCR5 (96.8%) while nearly 50% of cells also expressed α_4_β_7_, a cell surface protein that has been associated with CD4+ T-cell homing to the gut and may play a role in early events in HIV transmission (Figure 2A)[77]. Importantly, Choudhry, et al reported that increased CCR5 density is associated with decreased neutralization sensitivity in a well-characterized HeLa cell line system[52]. Consequently, we compared CD4, CCR5 and α_4_β_7_ receptor densities on the A3R5.7 line to TZM-bl and CD3/CD8 bi-specific antibody stimulated PBMC (Figure 2B). Mean CD4 density in both stimulated PBMC and TZM-bl (exceeded assay upper limit) was >2-fold higher than observed in the A3R5.7 line. Mean CCR5 density in the A3R5.7 line was nearly 6-fold higher than PBMC but 4-fold lower than TZM-bl. Mean α_4_β_7_ density in the A3R5.7 line was ~50% of that seen in PBMC while the TZM-bl was negative for α_4_β_7_. 

### Neutralization of tier 2 subtype B transmitted/founder IMC.LucR by sCD4 and mAbs

**Figure 3:**
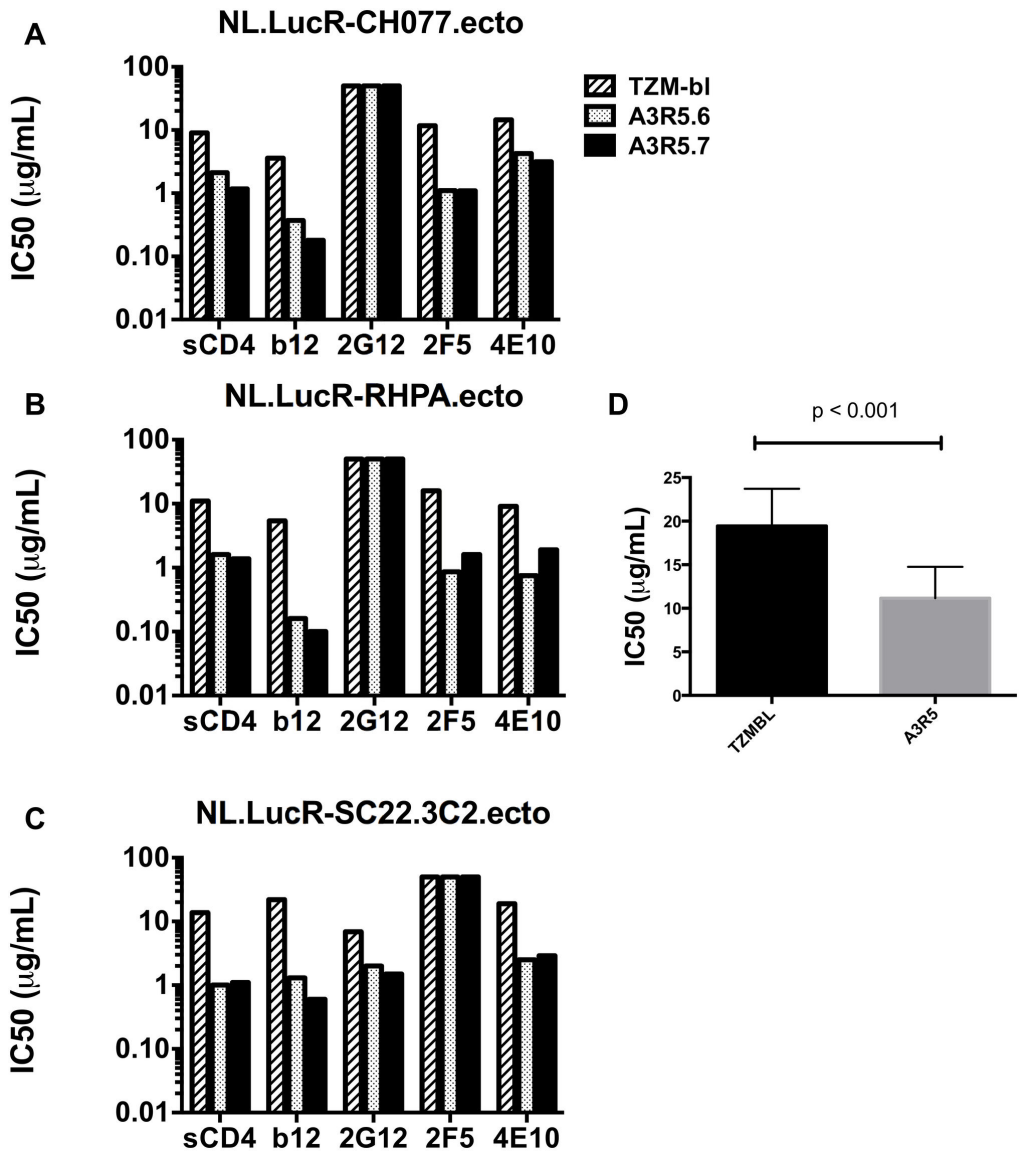
Subtype B neutralization in A3R5 cells and TZM-bl. **A**-**C**. **Neutralization sensitivity of three tier 2 subtype B IMC.LucR transmitted/founder viruses to sCD4 and a panel of four mAbs in two A3R5 cell lines and TZM-bl**. With the exception where no inhibition was noted for any cell line, the A3R5 cell lines showed greater neutralization for all inhibitors tested compared to TZM-bl. **D**. **Grouped inhibitor comparison between cell lines**. When grouped by cell line using the non-parametric Mann-Whitney test, there was a significant difference between the A3R5 cell lines and TZM-bl (Mann-Whitney test; p<0.001). IC50 titers are the concentration of inhibitor at which the Renilla luciferase signal (RLUs) was reduced by 50% compared to the virus control (no inhibitor). Inhibitor concentrations ranged from 0.78 μg/mL to 25 μg/mL. All assays were performed in the presence of DEAE-Dextran.

Potent neutralization of R5-tropic subtype B HIV-1 by sCD4 and mAbs has been well documented in mitogen-stimulated peripheral blood mononuclear cells (PBMC) [78–83]. However, there appear to be differences in measured neutralizing capacity when different cell types are used as targets [17,28,30,53,84]. To evaluate neutralization sensitivity in the A3R5.6 and .7 cell lines, sCD4 and the mAbs b12, 2G12, 2F5 and 4E10 were titered against three tier 2 subtype B transmitted/founder (T/F) IMC.LucR-Env.ecto viruses (NL.LucR-CH077.ecto, NL.LucR-RHPA.ecto and NL.LucR-SC32.3C2.ecto) and compared to inhibition in the TZM-bl line (Figure 3A-C)[71,72] and Edmonds et al., manuscript in preparation. Neutralization was equivalent or more potent in the A3R5 lines than in TZM-bl for every virus/inhibitor pair tested. There was no observed 2G12 mediated neutralization of NL.LucR-CH077.ecto and NL.LucR-RHPA.ecto while NL-LucR-SC32.3C2.ecto was insensitive to 2F5. When analyzed as a group, neutralization was significantly greater in the A3R5 cell lines compared to TZM-bl (Figure 3D)(Mann-Whitney test; p<0.001).

### Neutralization of chronic NL.LucR-CM235.ecto with sCD4, mAbs and HIV+ sera/plasma

**Figure 4:**
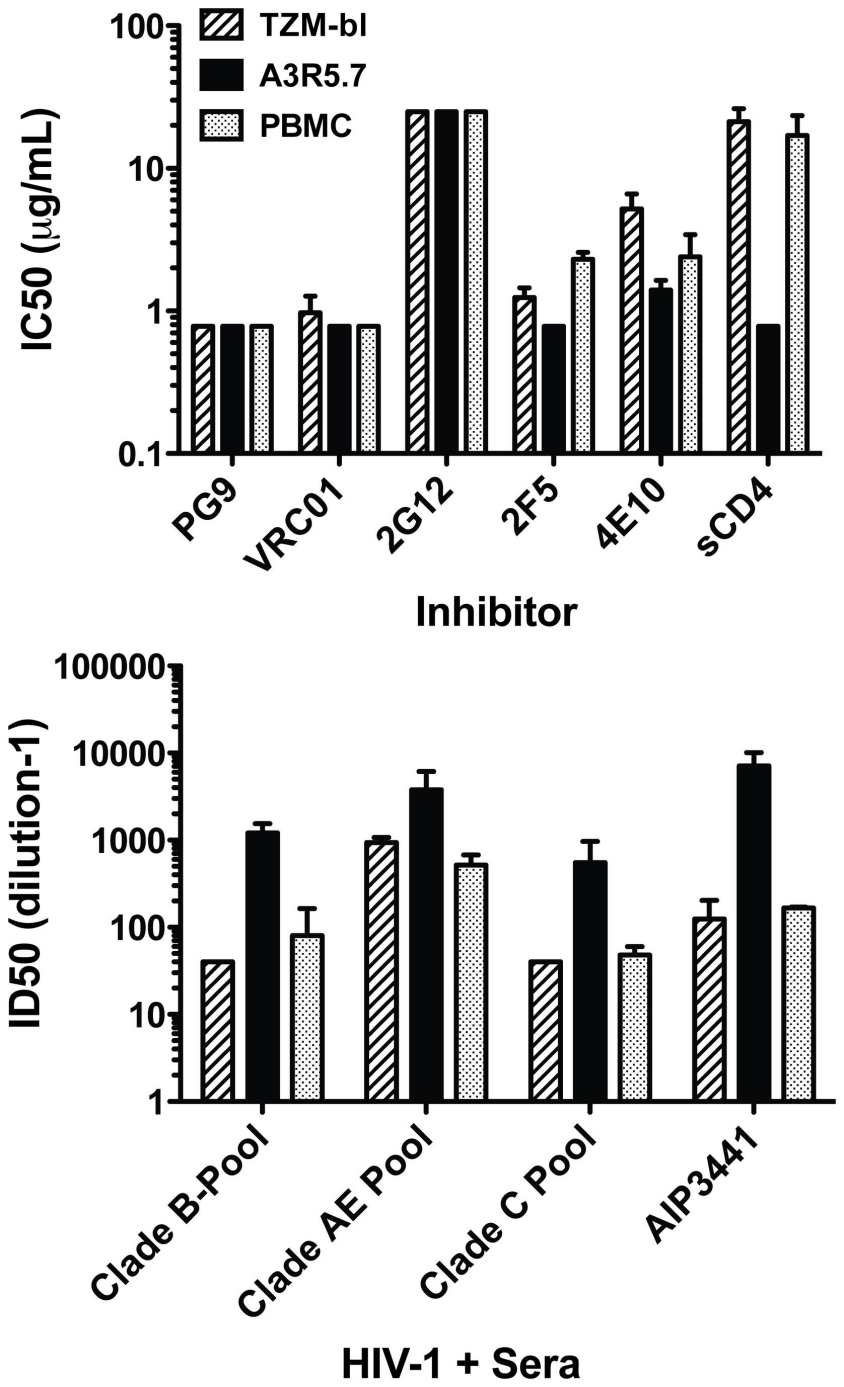
Neutralization of a tier 2 CRF_01 AE IMC.LucR virus in A3R5.7, TZM-bl and CD3.8 bi-specific antibody stimulated PBMC cell targets. **A**. **Neutralization with epitope-specific inhibitors**. NL.LucR-CM235.ecto was tested against a panel of monoclonal antibodies and sCD4 in each cell line. The highly potent PG9 ad VRC01 mAbs demonstrated IC50 titers below the limits of the assay (<0.78μg/mL). This chronic envelope IMC was insensitive to 2G12 (>25μg/mL) in all target cells. NL.LucR-CM235.ecto was more sensitive to the inhibitors 2F5, 4E10 and sCD4 in the A3R5.7 target cell compared to both TZM-bl and PBMC, reaching significance with sCD4 (Unpaired t-test with Welch correction; p<0.05). Inhibitors ranged from 0.78μg/mL to 25 μg/mL. **B**. **Neutralization with polyclonal sera**. NL.LucR-CM235.ecto was tested against a panel of pooled HIV-1 “+” sera/plasma and the individual AE-specific sera AIP3441 in each cell line. Sera dilutions ranged from 40-40,960. All sera appeared more potent in the A3R5.7 cell line compared to either TZM-bl or PBMC.

With the majority of new HIV-1 infections occurring outside the U.S and Europe, vaccine design has shifted toward non-subtype B immunogens to support ongoing human clinical trials occurring in Asia and Africa[1,85,86]. Consequently, we examined neutralization of an IMC.LucR-Env.ecto virus containing a chronic tier 2 CRF_01 AE envelope (NL.LucR-CM235.ecto) using a panel of mAbs, sCD4 (Figure 4A) and HIV+ sera/plasma from multiple clades (Figure 4B) in TZM-bl, A3R5.7 and CD3.8 bi-specific antibody stimulated PBMC[73,75,87]. As observed with subtype B in Figure 3, 2G12 failed to neutralize this virus in any of the target cells. The highly potent PG9 and VRC01 mAbs [29,32] neutralized this virus in all three cell targets at or near the lowest concentration tested (0.78 μg/mL)[29,32]. Neutralization by the gp41-specific 2F5 and 4E10 mAbs was slightly more potent in A3R5.7 cells than either TZM-bl or stimulated PBMC. However, the IC_50_ of the gp120 competitive inhibitor sCD4 was significantly lower in the A3R5.7 line (unpaired t-test; p<0.05) compared to both TZM-bl and PBMC. In Figure 4B, neutralization sensitivity to pooled sera/plasma from three different subtypes and from a subtype-matched (AE) HIV+ individual plasma was markedly greater in the A3R5.7 line versus TZM-bl and stimulated PBMC. However, the difference only reached significance (unpaired t-test; p<0.03) for the subtype B pool when comparing A3R5.7 vs. TZM-bl. 

### Neutralization of IMC.LucR viruses with subtype-matched HIV+ sera

**Figure 5:**
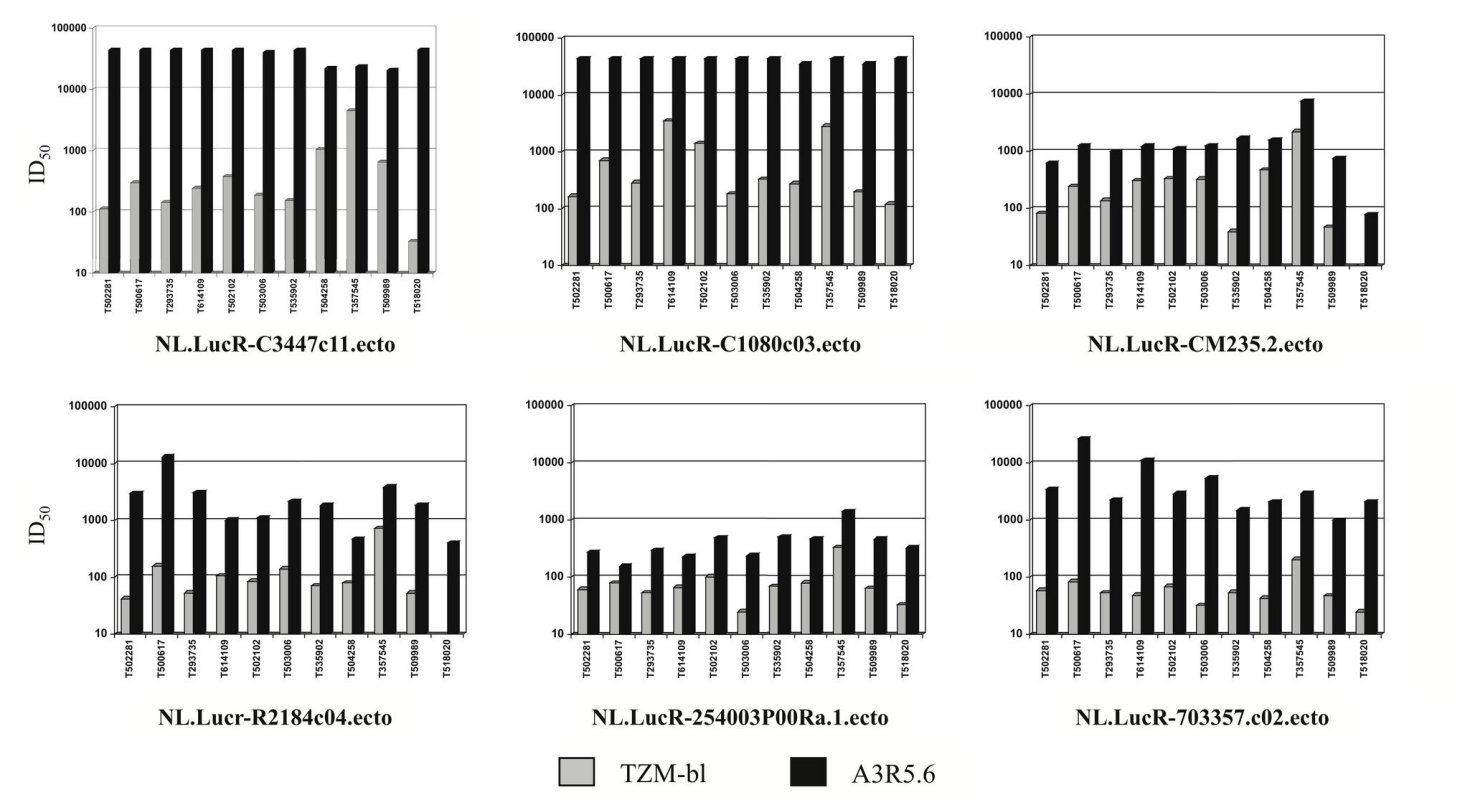
Comparison of neutralization sensitivity in A3R5 and TZM-bl cell lines. A panel of eleven HIV+ CRF_01 AE sera was assayed against two tier 1 (C3347.c11 and C1080.c03) and four tier 2 (CM235-2, R2184.c04, 254003P00Ra.1 and 703357.c02) CRR_01 AE Env.IMC.LucR viruses in the A3R5.6 and TZM-bl cell lines. ID50 titers are the dilution of serum at which the Renilla luciferase signal was reduced by 50% compared to the virus control (no serum). Neutralization sensitivity was significantly greater (p<0.001; Wilcoxon Rank Sum) in the A3R5 cell line compared to TZM-bl for all virus/sera pairs tested. Similar results were obtained with the A3R5.7 cell line.

To further investigate the enhanced sensitivity observed in A3R5 cells, we looked at the neutralization of five chronic envelope NL.LucR-AE.Env.ecto IMC (2 tier 1 and 3 tier 2), and one transmission/founder (T/F) full-length gp160 Env bearing CRF01_AE backbone IMC with a panel of eleven polyclonal sera from AE_CRF01 infected individuals. The ID_50_ titer for each virus/sera pair was greater in the A3R5.6 cell line compared to TZM-bl (Figure 5). Overall, the GMT against each virus was significantly higher in the A3R5.6 line (Wilcoxon Rank-Sum; p<0.001). Similar results were obtained in the A3R5.7 cell line (data not shown). 

## Discussion

The accurate assessment of humoral immune responses in HIV-1 vaccine recipients is key for the optimization of immunogen design, adjuvant choice and dosing regimen. Ultimately the study of NAbs has relevance for (1) the establishment of humoral correlates of protection that may facilitate the development of an effective vaccine, (2) in the identification of potential immunogens that induce desirable immune responses, or (3) in the development of novel monoclonal antibodies that may offer therapeutic or prophylactic proof of concept. To date a number of different assays have been used (summarized in Polonis et al. and Ochsenbauer & Kappes, in Current Opinions in HIV and AIDS, Vol 4, No 5, 2009, Montefiori and Mascola, editors); most share the same characteristics including a CD4+/CCR5+ target cell, HIV-1 infectious molecular clones, pseudoviruses or virus-like particles as the challenge, and a marker of infection such as p24 Gag or luciferase. The TZM-bl assay has been used widely in recent years [51,60,61] because of its ease of use, reproducibility, wide dynamic range, and low cost. While differences in sensitivity and specificity have been demonstrated between the TZM-bl and the more traditional PBMC neutralization assay [30,54,84,88,89], both have been used to identify correlates of protection in animal studies [90–92] and testing of HIV vaccine candidates in humans has not, to date, convincingly demonstrated the induction of broad Tier 2 Nab in either target[2,5,93]. 

A3R5 cells are T-lymphoblastoid in origin and naturally express CD4 and CXCR4 as well as the α_4_β_7_ integrin thought by some to be important in acute infection[77,94,95]. The A3R5 cell line was engineered to constitutively express CCR5 and demonstrates receptor density above stimulated CD4+ T cells but below that observed on TZM-bl cells. The relevance of this difference is currently unknown. 

Our findings suggest that the CD4+/CXCR4+/CCR5+ A3R5 T-cell line might be useful in the analysis of neutralization activity in HIV vaccine trials, especially in combination with recently developed panels of Env.IMC.LucR replication-competent reporter viruses[71,73](Edmonds et al., manuscript in preparation). In previous work, A3R5 lines have been utilized in the analysis of Nab from several HIV vaccine trials as well as the study of correlates of risk in RV144[2–4,87]. The cell line was found to be permissive to an extensive range of HIV-1 infectious molecular clones including those with envelopes from subtypes CRF01_AE and C that predominate in regions important for future HIV vaccine efficacy trials. Inhibition of subtype B and CRF01_AE IMC.LucR by sCD4, mAbs, pooled sera/plasma from multiple subtypes and subtype-matched individual HIV+ polyclonal sera appear to be greater in A3R5 cells than TZM-bls. In the context of a single chronic CRF01_AE IMC.LucR, this observation was extended to CD3.8 stimulated PBMC. The mechanism(s) underlying the observed disparity with the TZM-bl system remain to be elucidated. However, cell origin may play a role with regard to cell surface receptor density, cell membrane lipid composition, glycosylation patterns, and possibly virus entry mechanisms[52,53,61,96–102]. 

A recently published report by Montefiori et al. extends these observations to include enhanced neutralization in the A3R5 cell line in the context of two disparate HIV-negative vaccine studies performed in Thailand[2]. The first (Vax003) looked at injection drug users who received seven intramuscular inoculations of the AIDSVAX B/E gp120 protein[103]. At the time of peak NAb response (2 weeks post-4^th^ inoculation), strong tier 1 and occasional weak tier 2 neutralization was observed in the A3R5 assay, whereas weak tier 1 and no tier 2 neutralization were seen in the TZM-bl assay. The second trial (RV144) consisted of a large low-risk, heterosexual, community-based cohort vaccinated with an ALVAC prime (vCP1521) and AIDSVAX B/E boost[1]. While no tier 2 neutralization was recorded in either assay system, overall neutralization patterns suggested a higher level of sensitivity in the A3R5 line. The greater neutralization sensitivity observed in the A3R5 assay system versus the TZM-bl based assay was large enough in some cases to result in a reduction of the HIV IMC tier phenotype from 2 to 1 in the A3R5 line. This unexpected finding may prove important with regard to the selection of HIV IMC reagents used in support of future clinical trials. More comprehensive studies in appropriate HIV vaccine studies will be required to determine whether enhanced detection of neutralization observed with A3R5 cells will aid in the discovery of correlates of risk of infection.

Earlier neutralization work in A3R5 cells focused on vaccine-matched primary isolates adapted to growth in A3R5 cells. Utilizing these adapted viruses, 68% of vaccine recipients receiving the ALVAC-HIV vCP1521 prime and an AIDSVAX B/E boost had detectable antibody against the vaccine strain CM244 virus [3]. Importantly the data presented here show the utility of A3R5 to detect the neutralization of IMC encoding tier 2 CRF01_AE and subtype C and B Env, including IMC.LucR viruses engineered to express transmitter/founder (T/F) envelopes from different subtypes that are more closely related to sequences currently found in circulation[73](Edmonds et al., manuscript in preparation). 

More extensive characterization of neutralization sensitivity and specificity with panels of monoclonal antibodies and polyclonal sera in the context of standardized IMC panels in the A3R5 cell line is warranted given the pace of preclinical and clinical development of new Env immunogens (as monomer, trimer and vector-expressed). In this study, chimeric IMCs utilizing non-subtype B backbones and those utilizing NL4-3 backbones were used interchangeably, but continued study of these next-generation vectors may provide insight into how non-Env genes influence virus replication and sensitivity to neutralization[73]. Further, it would be useful to have A3R5 cells stably transduced with an LTR-driven marker, such as Luciferase, so that the extensive libraries of pseudoviruses created for the TZM-bl assay may be incorporated into current testing protocols. Ultimately the identification of a neutralizing antibody correlate of risk will further our understanding of HIV-1 infection and inform vaccine design.

## Supporting Information

Figure S1 **Subtype B primary isolate growth in A3R5 cell lines.**
(PDF)

Table S1 **IMC nomenclature and characteristics.**
(XLSX)
