# Supplementary material for: Detection of HIV-1 Neutralizing Antibodies in a Human CD4+/CXCR4+/CCR5+ T-Lymphoblastoid Cell Assay System
Source: PLoS One. 2013 Nov 28;8(11):e77756. doi: 10.1371/journal.pone.0077756 (PMC3842913; doi:10.1371/journal.pone.0077756)
Supplement: Figure S1 — Subtype B primary isolate growth in A3R5 cell lines. (PDF) [file pone.0077756.s001.pdf]

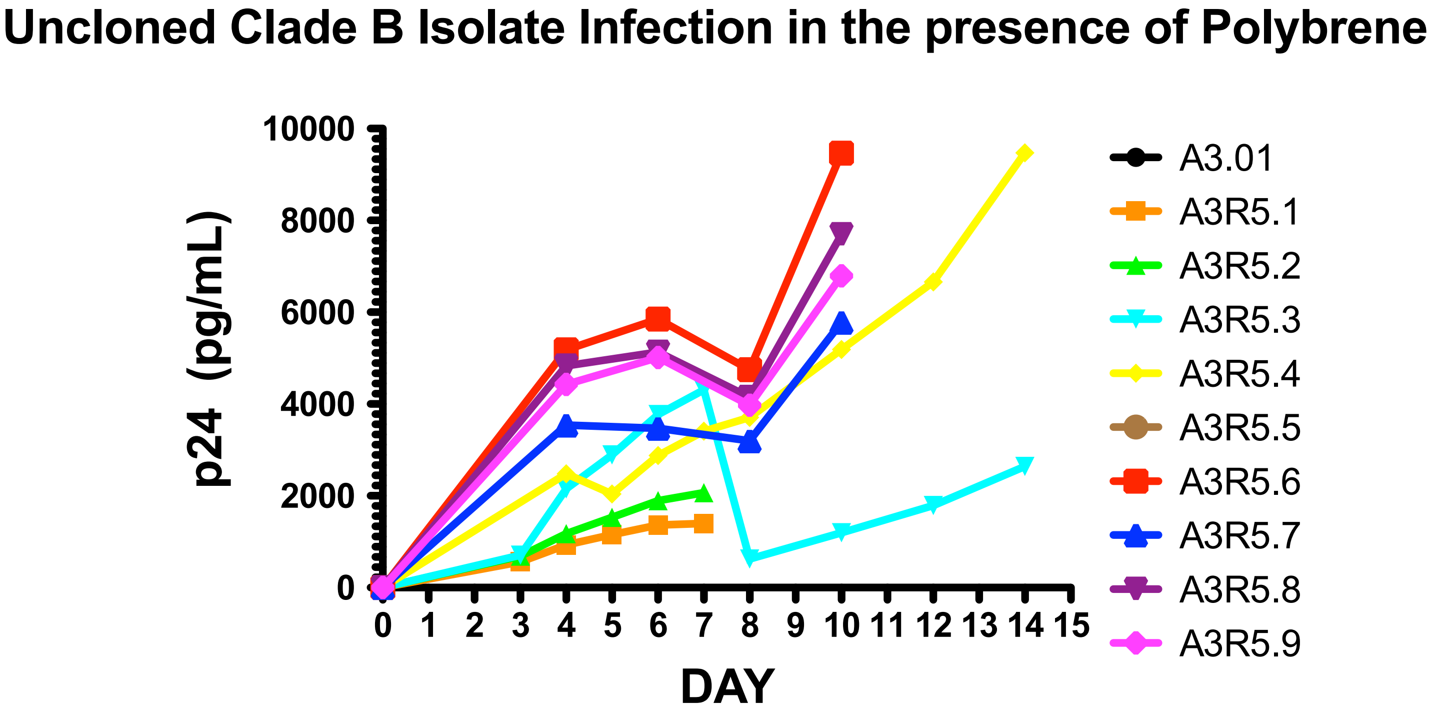

Supplementary Figure 1: Growth kinetics of US1(subtype B/R5) in each A3R5 cell line. Virus production was measured by p24 antigen in the supernatant using a commercial sandwich ELISA kit. Data are presented as the mean of six independent wells. Virus growth was negligible in the parent (non-R5 transfected) A3.01 cell line.
